# Cognitive predictors of daily activity performance

**DOI:** 10.1002/alz70857_107600

**Published:** 2025-12-26

**Authors:** Audrey A. Keleman, Alyssa Weakley, Rebecca M. Bollinger, Erin Foster, Nicole S. McKay, Peter R Millar, Jason J. Hassenstab, Tammie L.S. Benzinger, John C. Morris, Beau Ances, Susan L. Stark

**Affiliations:** ^1^ Washington University in St. Louis School of Medicine, St. Louis, MO, USA; ^2^ Department of Veterans Affairs, Aurora, CO, USA; ^3^ University of Colorado Denver, Aurora, CO, USA; ^4^ University of California, Davis School of Medicine, Sacramento, CA, USA; ^5^ Washington University in St. Louis, St. Louis, MO, USA; ^6^ Washington University in St. Louis, School of Medicine, St. Louis, MO, USA; ^7^ Washington University School of Medicine, St. Louis, MO, USA

## Abstract

**Background:**

This study aimed to investigate the relationship between cognition, Alzheimer's disease (AD) biomarkers, and instrumental activities of daily living (IADL) performance among cognitively normal (CN) older adults, to identify key predictors of IADL performance. A better understanding of the combined influence of cognitive function and AD pathology on IADL performance in CN older adults could lead to the development of more precise functional screening tools for preclinical AD, improved functional outcome measures for AD and aging, and the development of interventions designed to maintain or improve IADL performance.

**Method:**

CN older adults (*n* = 173) performed three IADL tasks (shopping, checkbook balancing, medication management) from the Performance Assessment of Self‐Care Skills in their home. Cognitive and AD biomarker assessments were completed in an academic medical center. Amyloid and tau positron emission tomography (PET) were used to measure AD pathology. IADL performance was transformed into one composite score and dichotomized (better vs. worse, split at median). Cognitive assessments were transformed to create composites: Global, Episodic Memory, Semantic Memory, Attention & Processing, Working Memory, and Visuospatial. Logistic regression models were used to examine cognitive and biomarker predictors of IADL performance while controlling for demographic covariates.

**Result:**

Cognitive domains, particularly the Global composite (OR=0.34, *p* < 0.001) and Attention & Processing (OR=0.40, *p* = 0.001), emerged as strong predictors of IADL performance. Higher amyloid burden (OR=1.82, *p* = 0.015) was associated with worse IADL performance and tauopathy (OR=1.61, *p* = 0.060) demonstrated a trend toward association. Comparison of predictive models identified additive influences of cognition and amyloid in predicting IADL performance.

**Conclusion:**

These findings suggest that cognitive function, particularly Global and Attention & Processing domains, and AD pathological burden play significant, complementary roles in IADL performance among CN older adults. Findings may be used to inform the development of sensitive functional assessments in preclinical AD that focus on key deficits, and later to develop interventions targeting important modifiable factors and compensatory rehabilitative methods to improve IADL performance in everyday life.

